# Focal Segmental Glomerulosclerosis Patient Baseline Characteristics in the Sparsentan Phase 3 DUPLEX Study

**DOI:** 10.1016/j.ekir.2024.01.032

**Published:** 2024-01-28

**Authors:** Howard Trachtman, Jai Radhakrishnan, Michelle N. Rheault, Charles E. Alpers, Jonathan Barratt, Hiddo J.L. Heerspink, Irene L. Noronha, Vlado Perkovic, Brad Rovin, Hernán Trimarchi, Muh Geot Wong, Alex Mercer, Jula Inrig, William Rote, Ed Murphy, Patricia W. Bedard, Sandra Roth, Stewart Bieler, Radko Komers

**Affiliations:** 1 Division of Nephrology, Department of Pediatrics, University of Michigan, Ann Arbor, Michigan, USA; 2 Division of Nephrology, Columbia University, New York, New York, USA; 3 Division of Pediatric Nephrology, University of Minnesota Medical School, Minneapolis, Minnesota, USA; 4 Department of Laboratory Medicine and Pathology, University of Washington, Seattle, Washington, USA; 5 Department of Cardiovascular Sciences, University of Leicester General Hospital, Leicester, UK; 6 Department of Clinical Pharmacy and Pharmacology, University of Medical Center Groningen, University of Groningen, Groningen, the Netherlands; 7 The George Institute for Global Health, Sydney, Australia; 8 Division of Nephrology, University of São Paulo School of Medicine, São Paulo, Brazil; 9 Faculty of Medicine, University of New South Wales Sydney, Sydney, New South Wales, Australia; 10 Division of Nephrology, Ohio State University Wexner Medical Center, Columbus, Ohio, USA; 11 Nephrology Service, Hospital Británico de Buenos Aires, Buenos Aires, Argentina; 12 Concord Clinical School, University of Sydney, Concord, New South Wales, Australia; 13 JAMCO Pharma Consulting, Stockholm, Sweden; 14 Travere Therapeutics Inc., San Diego, California, USA

**Keywords:** dual endothelin angiotensin receptor antagonist, focal segmental glomerulosclerosis, pediatric, race, randomized controlled clinical trial, sparsentan

## Abstract

**Introduction:**

The phase 3 DUPLEX trial is evaluating sparsentan, a novel, nonimmunosuppressive, single-molecule dual endothelin angiotensin receptor antagonist, in patients with focal segmental glomerulosclerosis (FSGS).

**Methods:**

DUPLEX (NCT03493685) is a global, multicenter, randomized, double-blind, parallel-group, active-controlled study evaluating the efficacy and safety of sparsentan 800 mg once daily versus irbesartan 300 mg once daily in patients aged 8 to 75 years (USA/UK) and 18 to 75 years (ex-USA/UK) weighing ≥20 kg with biopsy-proven FSGS or documented genetic mutation in a podocyte protein associated with FSGS, and urine protein-to-creatinine ratio (UP/C) ≥1.5 g/g. Baseline characteristics blinded to treatment allocation are reported descriptively.

**Results:**

The primary analysis population includes 371 patients (336 adult, 35 pediatric [<18 years]) who were randomized and received study drug (median age, 42 years). Patients were White (73.0%), Asian (13.2%), Black/African American (6.7%), or Other race (7.0%); and from North America (38.8%), Europe (36.1%), South America (12.7%), or Asia Pacific (12.4%). Baseline median UP/C was 3.0 g/g; 42.6% in nephrotic-range (UP/C >3.5 g/g [adults]; >2.0 g/g [pediatrics]). Patients were evenly distributed across estimated glomerular filtration rate (eGFR) categories corresponding to chronic kidney disease (CKD) stages 1 to 3b. Thirty-three patients (9.4% of 352 evaluable samples) had pathogenic or likely pathogenic (P/LP) variants of genes essential to podocyte structural integrity and function, 27 (7.7%) had P/LP collagen gene (*COL4A3/4/5*) variants, and 14 (4.0%) had high-risk *APOL1* genotypes.

**Conclusions:**

Patient enrollment in DUPLEX, the largest interventional study in FSGS to date, will enable important characterization of the treatment effect of sparsentan in a geographically broad and clinically diverse FSGS population.

## Graphical abstract

**Figure:**
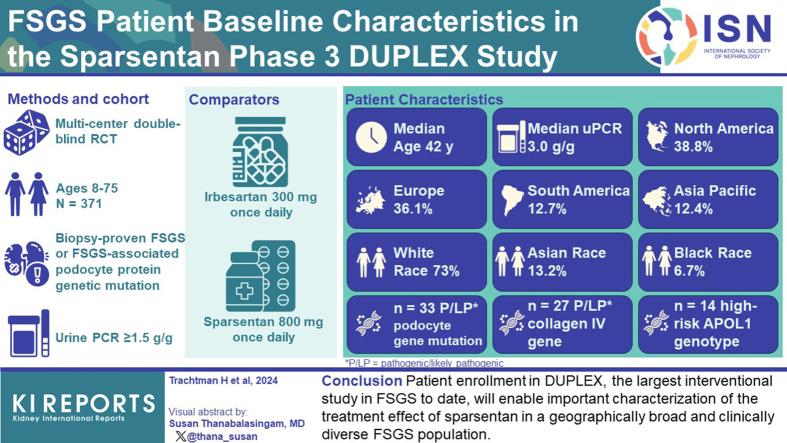

FSGS is a histological lesion characterized by segmental accumulation of glomerular extracellular matrix resulting in capillary obliteration and glomerular scarring.^1^ The prevalence of FSGS shows substantial variation according to racial background and geographic region.^2^^,^^3^ Patients with FSGS typically present with a variable degree of proteinuria and often nephrotic syndrome.^1^ A variety of heterogeneous clinical conditions may lead to FSGS-type lesions, which can be classified as primary, genetic, secondary, or FSGS of undetermined cause.^4^ Primary FSGS has no definitive cause,^1^ but it is presumed to be related to circulating factors that result in podocyte injury.^1^^,^^5^^,^^6^ Genetic causes include mutations in genes encoding proteins required for normal podocyte or glomerular basement membrane structure and/or function.^7^ Secondary causes of FSGS include drugs, infections, and conditions mediated by adaptive structural-functional responses to glomerular hypertension, hyperfiltration, or scarring due to remote episodes of active glomerulonephritis.^1^^,^^4^ FSGS of undetermined cause has been added as a subtype for patients who do not fit within the other categories.^4^ Regardless of etiology, patients with FSGS are at high risk for progression to kidney failure and the consequent need for dialysis and kidney transplant.^8^^,^^9^

Patients with FSGS are routinely treated with renin-angiotensin-aldosterone system inhibitors (RAASi), which are typically given in combination with immunosuppressive regimens in those with primary FSGS.^4^^,^^10^ Treatment with corticosteroids or other immunosuppressive agents aims to reduce proteinuria,^4^ an independent predictor of long-term kidney survival.^11^^,^^12^ However, the use of immunosuppressive therapy is limited by serious side effects, contraindications, the presence or development of treatment resistance and/or dependence, and a lack of response in patients with genetic FSGS.^4^^,^^13^ Therefore, there is an unmet need for more effective and tolerable treatments for patients with FSGS.

Endothelin type A receptor antagonists have demonstrated beneficial hemodynamic, anti-inflammatory, antifibrotic, and podocyte-protective effects in models of glomerular diseases.^14^ Additional benefits have been shown for the combination of RAASi and endothelin type A receptor antagonists in preclinical models^15^^,^^16^ and in patients with diabetic and nondiabetic nephropathies.^17^, ^18^, ^19^, ^20^

Sparsentan is a novel, nonimmunosuppressive, single-molecule, dual endothelin and angiotensin receptor antagonist with high selectivity for the endothelin type A receptor and angiotensin II subtype 1 receptor.^21^^,^^22^ Sparsentan was recently approved by the US Food and Drug Administration for the treatment of adults with IgA nephropathy at high risk of disease progression and is in development for the treatment of patients with FSGS. In the ongoing phase 2, randomized, double-blind, active-controlled DUET study, sparsentan demonstrated significantly greater antiproteinuric efficacy than irbesartan in patients with FSGS over an 8-week double-blind treatment period and was generally safe and well tolerated, with sustained proteinuria-lowering effects observed in an interim analysis of the open-label extension phase with a median follow-up of 4.6 years.^23^^,^^24^ The ongoing phase 3 DUPLEX trial was designed to evaluate the long-term antiproteinuric and nephroprotective effects and safety of sparsentan compared with the active control irbesartan in patients with FSGS.^25^ Here, we report the blinded baseline characteristics of patients enrolled in the DUPLEX trial, including subgroups defined by age (adult and pediatric), race (White, Asian, Black/African American, and Other), and geographic region (North America, Europe, Asia Pacific, and South America).

## Methods

### Study Design

The DUPLEX trial (EudraCT number: 2016-005141-23; US ClinicalTrials.gov Identifier: NCT03493685) is a phase 3, multicenter, international, randomized, double-blind, parallel-group, active-controlled trial designed to evaluate the efficacy and safety of sparsentan (initial dose of 400 mg daily for 2 weeks, titrated up to a target dose of 800 mg daily) compared with the angiotensin II receptor blocker irbesartan (initial dose of 150 mg daily for 2 weeks, titrated up to a target dose of 300 mg daily) in patients with FSGS. The study design has previously been reported^25^ and is briefly described below.

The study duration of 268 weeks includes a double-blind period of 112 weeks followed by an open-label extension period of up to 156 weeks. Eligible patients were male or female aged 8 to 75 years (United States and United Kingdom) or 18 to 75 years (outside the United States/United Kingdom) weighing ≥20 kg with biopsy-proven FSGS at any time in the past or documentation of a genetic mutation in a podocyte protein associated with FSGS, with UP/C (first morning void sample) of ≥1.5 g/g and eGFR ≥30 ml/min per 1.73 m^2^ at screening. Participants with a known secondary cause of FSGS or serological test findings diagnostic of another primary or secondary glomerular disease were excluded. Other key exclusion criteria were as follows: receipt of rituximab, cyclophosphamide, or abatacept within ≤3 months before screening (other chronic immunosuppressive medications must have stable dosage for ≥1 month before screening and during the screening period); a history of type 1 diabetes mellitus, uncontrolled type 2 diabetes mellitus (hemoglobin A1c >8%), or nonfasting blood glucose >180 mg/dl at screening; or significant cerebrovascular, cardiovascular, or hepatic conditions. Women who were pregnant, breastfeeding, or planning to become pregnant within the trial period were not allowed to enter the trial and frequent pregnancy testing was applied throughout the trial.

### Procedures

Patients on RAASi at screening underwent a 2-week washout period from these agents before randomization (day 1). Enrolled patients completed comprehensive baseline evaluations and clinical laboratory tests after screening (or after the washout period for patients on RAASi). During the double-blind period, clinic visits were scheduled at weeks 2, 4, 6, 8, 12, and then every 12 weeks thereafter. Routine blood and urine samples for laboratory assessments were analyzed at a central laboratory. eGFR at each visit was determined using the CKD Epidemiology formula (with race coefficient) for patients aged ≥16 years at screening and the modified Schwartz formula for patients aged <16 years at screening. UP/C was calculated based on an average of 3 first morning void urine samples, collected within 5 days before each visit. Safety evaluations included changes from baseline in body weight, vital signs, physical examinations, peripheral edema, 12-lead electrocardiogram, and clinical laboratory parameters, and the incidence of treatment-emergent adverse events.

### Endpoints and Assessments

The primary efficacy endpoint is the slope of eGFR over approximately 2 years of randomized treatment, evaluated following initiation of randomized treatment (total slope, from day 1 to week 108 in the USA) and following the initial acute effect of randomized treatment (chronic slope, from week 6 to week 108 in countries outside of the USA) according to regional regulatory requirements. Surrogate, secondary, and exploratory endpoints have been previously described.^25^

### Data Analysis

The blinded baseline characteristics of enrolled patients in the primary analysis population are descriptively summarized in this report. The primary analysis population includes all patients who were randomized and received at least 1 dose of the assigned study treatment.

### Genetic Testing and Variant Identification

Genomic DNA was extracted from peripheral blood samples collected at baseline. Genetic testing and variant identification was performed by a Clinical Laboratory Improvement Amendments-accredited laboratory (PreventionGenetics, LLC, Marshfield, WI) using a 73-gene nephrotic syndrome/FSGS panel (72-gene test panel plus *PODXL*, Supplementary Table S1). Next-generation sequencing was performed using PGxome, which covers the coding regions of targeted genes and ∼10 bases of noncoding DNA flanking each exon. Variant classification followed the American College of Medical Genetics and Genomics standards and guidelines.^26^ Individual variants were adjudicated by 4 qualified genetics experts and reported as pathogenic, likely pathogenic, or of uncertain significance (for variants essential to podocyte structure/function and for collagen variants); or as risk alleles (for *APOL1* variants). Each patient was further characterized based on the number, type, and form of inheritance of variants identified to assess whether the pathogenicity of the variant would likely be expressed. Genotyping was conducted in a manner that was blinded to investigators, patients, and the sponsor with respect to patient identification, treatment arms, and clinical response.

## Results

A total of 371 patients from 22 countries were enrolled, randomized, and received at least 1 dose of study drug, including 336 (90.6%) aged ≥18 years (adult population) and 35 (9.4%) aged <18 years (pediatric population). Enrolled patients were mainly from North America (38.8% of patients) and Europe (36.1%), followed by South America (12.7%) and the Asia Pacific region (12.4%). The median (interquartile range) age of the overall population was 42.0 (27.0–56.0) years; 53.9% were male; most were White (73.0%); and 21.3% were of Hispanic or Latino ethnicity (Table 1). The median age at diagnosis was 37.0 years (interquartile range, 23.0–51.0), and the median time from diagnosis to enrollment was 2.0 years (1.0–6.0). A history of nephrotic syndrome was documented in 30.2% of patients, and 64.2% had a history of hypertension. Mean systolic/diastolic blood pressure at screening was 128.0/81.1 mm Hg and at baseline—after the 2-week RAASi washout—was 131.9/83.8 mm Hg. Edema was present at baseline in 38.0% of patients (Table 2).

**Table 1:** Baseline demographic characteristics and relevant medical history

Characteristics	All patients (*N* = 371)	Adult patients (*n* = 336)	Pediatric patients (*n* = 35)
Age at informed consent, years			
Median (IQR)	42.0 (27.0–56.0)	44.0 (33.0–57.0)	14.0 (12.0–16.0)
Mean ± SD	41.6 ± 16.9	44.5 ± 15.1	14.1 ± 2.3
Sex			
Male	200 (53.9)	188 (56.0)	12 (34.3)
Female	171 (46.1)	148 (44.0)	23 (65.7)
Race^a^			
White	271 (73.0)	246 (73.2)	25 (71.4)
Asian	49 (13.2)	48 (14.3)	1 (2.9)
Black or African American	25 (6.7)	21 (6.3)	4 (11.4)
Other	26 (7.0)	21 (6.3)	5 (14.3)
Ethnicity			
Not Hispanic or Latino	281 (75.7)	260 (77.4)	21 (60.0)
Hispanic or Latino	79 (21.3)	65 (19.3)	14 (40.0)
Not reported	7 (1.9)	7 (2.1)	0 (0.0)
Unknown	4 (1.1)	4 (1.2)	0 (0.0)
Age at FSGS diagnosis, years^b^	37.0 (23.0–51.0)	40.0 (27.0–53.0)	12.5 (10.0–15.0)
Time from FSGS diagnosis to informed consent, yrs^c^	2.0 (1.0–6.0)	2.0 (1.0–6.0)	2.0 (1.0–5.0)
History of diabetes and impaired fasting glucose	58 (15.6)	58 (17.3)	0 (0.0)
HbA1c, all patients, mean ± SD	5.5 ± 0.6	5.5 ± 0.6	5.1 ± 0.4
HbA1c, patients with diabetes or impaired fasting glucose, mean ± SD	6.4 ± 0.8	6.4 ± 0.8	n/a
History of hypertension	238 (64.2)	227 (67.6)	11 (31.4)
Blood pressure, mm Hg, mean ± SD			
Systolic	131.9 ± 14.9	133.4 ± 14.4	117.4 ± 12.5
Diastolic	83.8 ± 10.5	84.7 ± 10.2	75.7 ± 10.4
BMI, kg/m^2^, mean ± SD	27.7 ± 5.9	28.0 ± 5.7	24.9 ± 7.3
Documented history of nephrotic syndrome^d^	112 (30.2)	91 (27.1)	21 (60.0)
Baseline nephrotic syndrome^e^	54 (14.6)	41 (12.2)	13 (37.1)

BMI, body mass index; FSGS, focal segmental glomerulosclerosis; HbA1c, hemoglobin A1c; IQR, interquartile range; n/a, not applicable.

Data are given as *n* (%) or median (IQR) unless otherwise noted.

a Patients who selected more than 1 race are included in Other. “Other” race included American Indian or Alaska Native, Native Hawaiian or Other Pacific Islander, and Other.

b Age at FSGS diagnosis is derived based on the year of FSGS diagnosis and year of birth.

c Time from FSGS diagnosis is derived based on the year of FSGS diagnosis and year of signed informed consent.

d A patient was considered to have documented history of nephrotic syndrome if the term “Nephrotic syndrome” was present in their medical history or if all of the following conditions were met at any of the visits before the first dose of randomized treatment: UP/C >3.5 g/g (adults) or UP/C >2 g/g (pediatrics), serum albumin <3.0 g/dl, and abnormal edema from physical examination.

e Defined as patients with all of the following before first dose: UP/C >3.5 g/g (adults) or >2 g/g (pediatrics); serum albumin <3.0 g/dl; and abnormal edema from physical examination.

**Table 2:** Edema at baseline

Characteristic	All patients (*N* = 371)	Adult patients (*n* = 336)	Pediatric patients (*n* = 35)
Edema present	141 (38.0)	130 (38.7)	11 (31.4)
Edema grade			
0	230 (62.0)	206 (61.3)	24 (68.6)
1	92 (24.8)	85 (25.3)	7 (20.0)
2	34 (9.2)	32 (9.5)	2 (5.7)
3	15 (4.0)	13 (3.9)	2 (5.7)
4	0 (0.0)	0 (0.0)	0 (0.0)

Data are given as *n* (%).

### Laboratory Assessments

Median UP/C at baseline was 3.0 g/g, and 42.6% of patients had nephrotic range UP/C (Table 3). Mean (range) eGFR at baseline was 63.8 (21–208) ml/min per 1.73 m^2^, with a broad distribution of patients across eGFR categories corresponding to CKD stage 1 (18.9%), stage 2 (26.4%), stage 3a (21.3%), and stage 3b (27.2%); 23 patients (6.2%) met the criterion of an eGFR of >30 ml/min per 1.73 m^2^ at screening but fell to <30 (but ≥15) ml/min per 1.73 m^2^ (CKD stage 4) at the time of baseline measurement (day 1/randomization). Other laboratory tests showed values largely within the normal ranges (≥75% of patients within normal range), except for elevations in total cholesterol (63.6% of patients), low-density lipoprotein cholesterol (46.4% of patients), triglycerides (63.6% of patients), serum creatinine (70.1% of patients), and serum cystatin C (74.4% of patients), and lower than normal values for serum albumin (42.0% of patients). No patients had alanine aminotransferase elevations >3× above normal, and 1 patient (0.3%) had aspartate aminotransferase >3× above normal.

**Table 3:** Laboratory values at baseline

Characteristics	All patients (*N* = 371)	Adult patients (*n* = 336)	Pediatric patients (*n* = 35)
UP/C, g/g	3.0 (2.2–4.6)	2.9 (2.1–4.1)	5.0 (3.3–7.3)
Nephrotic range UP/C (>3.5 g/g in adults; >2.0 g/g in pediatric patients)	158 (42.6)	124 (36.9)	34 (97.1)
UA/C, g/g	2.5 (1.7–3.5)	2.4 (1.7–3.3)	3.6 (2.7–4.6)
eGFR,^a^ ml/min per 1.73 m^2^			
Mean ± SD	63.8±30.3	61.1±27.0	89.6±45.1
Median (IQR)	55.0 (41.0–80.0)	53.0 (40.5–78.0)	79.0 (53.0–124.0)
Range	21–208	21–146	31–208
eGFR			
≥90 ml/min per 1.73 m^2^	70 (18.9)	58 (17.3)	12 (34.3)
≥60 to <90 ml/min per 1.73 m^2^	98 (26.4)	86 (25.6)	12 (34.3)
≥45 to <60 ml/min per 1.73 m^2^	79 (21.3)	74 (22.0)	5 (14.3)
≥30 to <45 ml/min per 1.73 m^2^	101 (27.2)	95 (28.3)	6 (17.1)
≥15 to <30 ml/min per 1.73 m^2^	23 (6.2)	23 (6.8)	0 (0.0)
Hemoglobin, g/l, mean ± SD	134.4±18.1	134.8±18.5	131.0±13.9
Plasma lipid profile, mmol/l, mean ± SD			
Total cholesterol	6.2±2.4	6.1±2.3	7.3±3.1
HDL-C	1.5±0.5	1.5±0.5	1.3±0.5
LDL-C	3.6±1.9	3.5±1.9	4.5±2.3
Triglycerides	2.3±1.6	2.3±1.4	3.1±2.6
Serum albumin, g/l			
Mean ± SD	34.9±7.4	35.6±7.0	28.3±8.1
Median (IQR)	36.0 (30.0–40.0)	37.0 (31.0–41.0)	29.0 (22.0–35.0)
Serum creatinine, μmol/l, mean ± SD	124.5±49.3	128.1±48.2	89.9±47.5
Serum potassium, mmol/l, mean ± SD	4.3±0.5	4.3±0.5	4.3±0.4
ALT, U/l	20.7±12.6	21.1±12.8	16.5±9.3
AST, U/l	22.1±10.6	22.0±10.5	22.2±11.7

CKD-EPI, CKD Epidemiology Collaboration; eGFR, estimated glomerular filtration rate; HDL-C, high-density lipoprotein cholesterol; IQR, interquartile range; LDL-C, low-density lipoprotein cholesterol; UA/C, urine albumin-to-creatinine; UP/C, urine protein-to-creatinine ratio

Data are given as *n* (%), median (IQR), or mean±SD unless otherwise noted.

a eGFR was determined using the CKD-EPI equation for patients ≥16 years of age at screening, and the modified Schwartz formula for patients <16 years of age at screening.

### Genetic Variants

**Figure 1:**
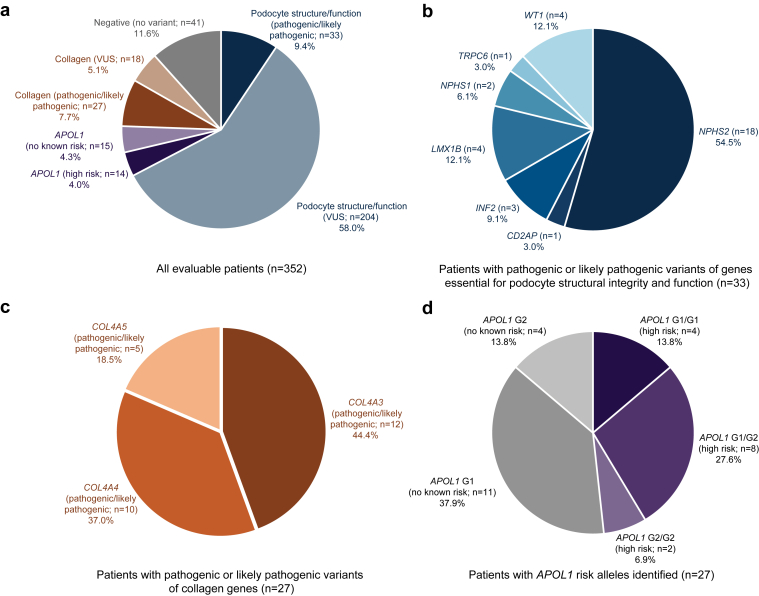
Percentage of patients with (a) each class of variant among all evaluable patients (*n* = 352), (b) genes essential for podocyte structural integrity and function identified as pathogenic or likely pathogenic variants (*n* = 33), (c) collagen gene variants identified as pathogenic or likely pathogenic (*n* = 27), and (d) *APOL1* gene variants identified (*n* = 29). Among the 371 patients in the DUPLEX primary analysis population, a total of 352 patients were included in the genetic variant evaluable population (9 patients did not provide consent for genetic testing, 2 samples were not shipped according to Q2 manifest, 5 samples had inadequate sample volume, and 3 patients with Trisomy X or mosaic copy gain were excluded). VUS, variant of unknown significance.

Overall, 33 patients (9.4% of the 352 evaluable samples) were identified as having P/LP variants of genes essential to podocyte structural integrity and function (*NPHS2*, *CD2AP*, *INF2*, *LMX1B*, *NPHS1*, *TRPC6*, and *WT1*; Figure 1a and b). An additional 27 patients (7.7% of evaluable) had P/LP collagen gene (*COL4A3*, *4*, and *5*) variants (Figure 1c), and 14 patients (4.0%) were identified as having *APOL1* high-risk (G1/G1, G1/G2, or G2/G2) variants (Figure 1d). Patients with high-risk *APOL1* genotypes identified as Black/African American (9/14) or selected “other” (5/14) in the electronic case report form (i.e., race other than White, Black or African American, Asian, American Indian or Alaska Native, or Native Hawaiian or Other Pacific Islander). The remainder of evaluable patients had no kidney disease-associated variants detected (11.6%) or variants of uncertain significance (67.3%) (Figure 1a).

### Pretreatment and Baseline Medications

In total, 73.0% of patients received a RAASi in the pretreatment period before the first dose of study medication (i.e., before the 2-week washout) (Table 4). At baseline and continuing into the double-blind period, 59.0% of patients were receiving non-RAASi antihypertensive medications, including diuretics; 58.5% were receiving lipid-lowering medications; and 24.8% were receiving immunosuppressives (14.3% steroids, 14.6% calcineurin inhibitors, 0.5% adrenocorticotropic hormone, 0.3% other).

**Table 4:** Pretreatment and baseline medications

Characteristics	All patients (*N* = 371)	Adult patients (*n* = 336)	Pediatric patients (*n* = 35)
Pretreatment RAASi^a^			
Any RAASi	271 (73.0)	253 (75.3)	18 (51.4)
ACEi	133 (35.9)	119 (35.4)	14 (40.0)
ARB	163 (43.9)	157 (46.7)	6 (17.1)
Aldosterone blockers	22 (5.9)	22 (6.5)	0 (0)
Baseline medication use^b^			
Non-RAASi antihypertensive medications	219 (59.0)	206 (61.3)	13 (37.1)
Diuretics	131 (35.3)	121 (36.0)	10 (28.6)
Beta-blockers	90 (24.3)	88 (26.2)	2 (5.7)
Calcium channel blockers	114 (30.7)	107 (31.9)	7 (20.0)
Alpha-blockers	49 (13.2)	48 (14.3)	1 (2.9)
Other	22 (5.9)	22 (6.6)	0 (0)
≥2 antihypertensive medications at baseline	124 (33.4)	116 (34.5)	8 (22.9)
Number of antihypertensive medications per patient			
Mean ± SD	1.9±1.0	1.9±1.0	1.7±0.6
Median (IQR)	2.00 (1–2)	2.00 (1–2)	2.00 (1–2)
RAASi^c^	12 (3.2)	12 (3.6)	0 (0)
Lipid-lowering medications	217 (58.5)	205 (61.0)	12 (34.3)
Immunosuppressive agents^d^			
Any immunosuppressive agents	92 (24.8)	82 (24.4)	10 (28.6)
Steroids	53 (14.3)	50 (14.9)	3 (8.6)
CNIs (cyclosporine, tacrolimus)	54 (14.6)	45 (13.4)	9 (25.7)
ACTH	2 (0.5)	2 (0.60)	0 (0)
Other	1 (0.3)	1 (0.30)	0 (0)

ACEi, angiotensin converting enzyme inhibitor; ACTH, adrenocorticotropic hormone; ARB, angiotensin receptor blocker; CNI, calcineurin inhibitor; IQR, interquartile range; RAS, renin-angiotensin system; RAASi, renin-angiotensin-aldosterone system inhibitors.

Data are given as n (%) unless otherwise noted.

a Pretreatment medications were initiated and stopped before the initial dose of study medication.

b Baseline medications were started before randomization (Day 1) and continued after the initial dose of study medication.

c 12 patients were on RAASi at baseline, of whom 8 were on RAS inhibitors and 4 were on aldosterone antagonists.

d Only immunosuppressive agents for renal indications are included. No patients received mycophenolate mofetil plus azathioprine.

### Baseline Characteristics in the Adult and Pediatric Populations

The median age of adult (*n* = 336) and pediatric (*n* = 35) patients in DUPLEX was 44.0 and 14.0 years, respectively. Several notable numerical differences were observed between age groups, including a higher proportion of Black/African American patients, a lower proportion of Asian patients, and a higher proportion of patients with a documented history of nephrotic syndrome in the pediatric group compared with the adult group (Table 1). Pediatric patients had higher mean eGFR and more frequently had nephrotic range UP/C at baseline compared with adults (Table 3). Compared with adults, a lower percentage of pediatric patients were receiving RAASi before the first dose of study medication, non-RAASi antihypertensive medications (including diuretics) at baseline, and lipid-lowering medications at baseline, and a higher percentage of pediatric patients were receiving immunosuppressive agents at baseline (Table 4).

### Baseline Characteristics by Racial Background

Considering the literature demonstrating differences in the prevalence of FSGS according to racial background and geographic region,^2^^,^^3^ baseline characteristics were compared between White (*n* = 271), Asian (*n* = 49), Black/African American (*n* = 25), and Other race (*n* = 26) subgroups (Supplementary Tables S2 and S3). Several notable numerical differences were observed between race subgroups, including a higher percentage of female patients, younger age (at informed consent and at diagnosis), and higher mean eGFR in the Black/African American subgroup compared with other race subgroups. Asian patients more frequently had a history of diabetes or impaired fasting glucose, and White patients more frequently had nephrotic range UP/C, compared with other race subgroups.

### Baseline Characteristics by Geographic Region

Baseline characteristics were further compared by geographic region of enrollment as follows: North America (*n* = 144), Europe (*n* = 134), South America (*n* = 47), and Asia Pacific (*n* = 46; Supplementary Tables S4 and S5). Several notable numerical differences were observed between geographic regions, including a younger age at both informed consent and diagnosis among patients from North America (only sites in the USA and then United Kingdom could enroll patients aged <18 years), a higher proportion of patients with a history of diabetes or impaired fasting glucose among patients from the Asia Pacific region, and a higher proportion of patients with edema among those from South America, when compared with other regions.

## Discussion

A lack of large interventional trials has impeded the development of new therapies for patients with FSGS. DUPLEX is the largest international, randomized controlled trial in patients with FSGS to date, and employed an active control arm—the angiotensin II receptor blocker irbesartan—that is consistent with treatment guideline recommendations in FSGS.^4^ Here, we report baseline characteristics and genetics for the 371 patients with FSGS enrolled in the DUPLEX trial of sparsentan. Despite receiving available standard of care, including immunosuppressive therapy and antihypertensive agents, 43% of patients in DUPLEX had nephrotic range proteinuria at baseline. The high-risk demographics in this patient population should be taken into consideration when evaluating the final results of the DUPLEX trial and the high unmet need for safe and effective treatment options in patients with FSGS.

The DUPLEX trial enrolled patients with FSGS (excluding those with a known secondary cause of FSGS) and UP/C ≥1.5 g/g at screening. A total of 33 patients (9% of the 352 evaluated) were identified as having pathogenic, or likely pathogenic, genetic variants associated with monogenic forms of genetic FSGS. Most of the P/LP variants affected genes associated with slit diaphragm proteins, with 18 patients (5% of patients tested) with the pathogenic variants affecting the gene encoding podocin (*NPHS2*).

Several recent studies have explored the prevalence of monogenic causes of steroid-resistant nephrotic syndrome and/or FSGS. Among patients with early-onset steroid-resistant nephrotic syndrome, a causative mutation was identified in 25% of families in an international cohort^27^ and in 23.6% of patients in the pediatric PodoNet international registry (22% among patients with a histopathologic diagnosis of FSGS).^28^ *NPHS1* and *NPHS2* were among the most commonly affected genes in both cohorts.^27^^,^^28^ A causative gene for FSGS was identified in 11% of adults with FSGS in the Toronto GN registry^29^ and 42.9% of adults with FSGS in a single-center analysis in the USA.^30^

In addition to mutations in genes coding for proteins essential to podocyte structural integrity and function, recent studies have identified a high prevalence of *COL4A3/4/5* variants in patients with FSGS^29^^,^^30^ and in patients with CKD in general.^31^ These variants were common in DUPLEX (detected in 8% of evaluated patients), in accordance with previously mentioned studies. Mutations in these type IV collagen genes result in a diverse clinical spectrum ranging from isolated microhematuria to early-onset kidney failure with extra-renal involvement.^32^ Alport syndrome, a type IV collagen-associated nephropathy, can manifest histologically with different glomerular patterns of injury, one of which is FSGS.^33^^,^^34^ In a large exome sequencing study of patients with CKD, more than half of patients with *COL4A3/4/5* variants did not have a clinical diagnosis of the classically associated nephropathies (Alport syndrome or thin basement membrane disease), which led to reclassification of the disease in some patients.^31^ These findings highlight the need for more broad use of genetic testing for accurate diagnosis of kidney diseases. Patients with genetic FSGS are unlikely to respond to immunosuppression and represent a patient population with high unmet need.^4^ DUPLEX is the largest FSGS trial to incorporate genotype testing to date and will allow for evaluation of sparsentan efficacy in patients with a variety of mutations underlying FSGS for whom plausible treatment options are currently lacking.

Baseline patient characteristics by race and geographic region for the DUPLEX study population may provide insight into potential differences in FSGS presentation. This is of particular interest considering the established risk of FSGS in African American patients harboring *APOL1* risk alleles,^1^^,^^3^^,^^35^ and the faster rate of kidney disease progression observed among patients with CKD^36^ or FSGS^37^ with a high-risk compared to a low-risk *APOL1* genotype. Four percent (*n* = 14) of the evaluable DUPLEX population had a high-risk *APOL1* genotype, defined as having the G1/G1, G1/G2, or G2/G2 genotype,^36^ the majority of whom identified as Black or African American. Targeted inhibition of APOL1 channel function reduced proteinuria in patients with biopsy-proven FSGS and a high-risk *APOL1* genotype in a small phase 2 study, providing evidence that toxic gain-of-function *APOL1* variants contribute to proteinuric CKD.^38^ In DUPLEX, Black/African American patients and those from North America were the youngest among the race subgroups and geographic region subgroups, respectively. This is likely due to the enrollment methods used in DUPLEX (children were not enrolled outside the USA/UK, and most Black/African American patients were enrolled in North America). Black/African American patients in DUPLEX had numerically higher mean eGFR, and White patients more frequently had nephrotic range UP/C compared with other race subgroups. Reasons for these apparent differences are unclear; however, these comparisons are limited by the small size of some subgroups. Asian patients and those from the Asia Pacific region in DUPLEX most frequently had a history of diabetes or impaired fasting glucose; this is consistent with findings at the US population level, which may reflect a higher risk for diabetes among Asian versus White individuals.^39^^,^^40^

Several features of the DUPLEX trial design and patient population distinguish it from previous trials, such as the FSGS Clinical Trial (FSGS-CT) and the dapagliflozin and prevention of adverse outcomes in CKD (DAPA-CKD) trial.^41^^,^^42^ The FSGS-CT trial compared a 12-month course of cyclosporine to dexamethasone plus mycophenolate mofetil in 138 patients with steroid-resistant FSGS aged 2 to 40 years.^41^ Unlike DUPLEX, all patients in FSGS-CT were steroid-resistant; and patients in FSGS-CT were more frequently pediatric (67.4% vs. 9.4%) or Black (37.9% vs. 7.8%) and were more likely to have higher proteinuria (median UP/C 4.0 vs. 3.0 g/g) at baseline compared with patients in DUPLEX.^41^^,^^43^ The placebo-controlled DAPA-CKD trial evaluated the effects of the SGLT2 inhibitor, dapagliflozin on renal and cardiovascular outcomes in adults with diabetic and nondiabetic CKD, including 104 patients with biopsy-confirmed FSGS.^42^ The FSGS cohort of the DAPA-CKD trial was older (mean age 54.0 vs. 41.6 years) and had a higher proportion of males (67.3% vs. 53.9%) and Asian patients (28.8% vs. 13.5%), as well as lower baseline mean eGFR (41.9 vs. 63.8 ml/min per 1.73 m^2^) than the DUPLEX study population.^42^ Unlike DUPLEX, the DAPA-CKD trial permitted patients with secondary causes of FSGS and excluded pediatric patients and those on chronic immunosuppression.^42^^,^^44^ In addition to the differences in patient population between trials, the active comparator in DUPLEX is a head-to-head comparison with maximum labeled or tolerated irbesartan, which is known to have beneficial renal effects such as improved creatinine clearance and decreased protein excretion,^45^ whereas the control arm in FSGS-CT was an immunosuppressive regimen and DAPA-CKD used a placebo comparator on background RAASi treatment without a protocol requirement to titrate to a maximal tolerated dose.^41^^,^^42^

Although the number of pediatric patients enrolled in DUPLEX was relatively small, several notable differences were observed when comparing the pediatric and adult populations enrolled in DUPLEX. These age group differences align with an analysis of 3 independent North American-based FSGS cohorts, including a prospective cohort study (NEPTUNE), a randomized controlled trial (FSGS-CT), and a registry (Kidney Research Network) and may inform differences in disease presentation according to age.^43^ Pediatric patients consistently had higher baseline eGFR, more frequently had nephrotic syndrome, and were less frequently administered RAASi than adult patients across DUPLEX and the 3 North American studies. These findings are not surprising, given that pediatric patients were likely biopsied earlier in the course of the disease and present more often with glomerular hyperfiltration than adult patients. In addition, immunosuppressive therapy at baseline was more common in pediatric patients compared with adults in DUPLEX and in the NEPTUNE and Kidney Research Network patient cohorts.^43^ Although new treatments with nephroprotective effects are needed for all patients with FSGS, this is a particularly high unmet need in the pediatric population for whom there is a paucity of randomized controlled trial data. A subgroup analysis of the randomized, double-blind, active-controlled phase 2 DUET study indicated strong antiproteinuric effects of sparsentan within both the adult and pediatric patient populations.^23^ The DUPLEX study will provide long-term data to further characterize the treatment effects of sparsentan, including adult and pediatric patients with a variety of mutations underlying FSGS.

A limitation of the DUPLEX study is the lack of available central biopsy reading at enrollment. The proportion of Black/African American patients enrolled in DUPLEX was relatively low despite specific measures to facilitate recruitment of Black/African American patients, including establishment of a cross-functional diversity team; collaboration with Black Health Matters to develop an FSGS and Clinical Trial Awareness Program for the African American population; engagement with a contract research organization to facilitate local outreach; and communications with sites, national leaders, and the steering committee to emphasize recruitment of diverse populations. Enrollment was also limited among other groups, such as those from East/South Asia and South America. Data were blinded in this analysis of patient baseline characteristics, which did not permit comparisons of sparsentan versus irbesartan groups and may have resulted in an overestimation of the percentage of patients with genetic variants due to the possibility of more than 1 mutation per patient. In addition, subgroup comparisons should be interpreted with caution due to the small number of patients in some groups.

## Conclusion

Sparsentan is a novel, nonimmunosuppressive, single-molecule dual endothelin and angiotensin II receptor antagonist. It is being evaluated in the DUPLEX trial as a promising new treatment for FSGS. DUPLEX enrolled adult and pediatric patients with biopsy-confirmed FSGS or a documented genetic mutation in a podocyte protein associated with FSGS (excluding FSGS with a known secondary cause) and at high risk of progression to kidney failure. The enrollment of both adult and pediatric patients across differing racial backgrounds, geographic regions, and CKD stages will enable characterization of the treatment effect of sparsentan in a geographically broad and clinically diverse FSGS population.

## Disclosure

HTra has served as a consultant to and/or a member of a data monitoring committee for Aclipse, Akebia, Boehringer-Ingelheim, ChemoCentryx, Eloxx Pharmaceuticals, Goldfinch Bio, Inc., Maze Therapeutics, Inc., Natera, Otsuka, PhaseV, Travere Therapeutics, Inc., and Walden. JR reports consulting fees from Calliditas, Chinook, and Travere Therapeutics; and grant support from Travere Therapeutics. MNR has served as clinical trial site PI for Chinook, Kaneka, Reata, River 3 Renal Corp., Sanofi, and Travere Therapeutics, Inc.; consultant to Visterra; and Data and Safety Monitoring Board member for Advicenne. CEA has served as consultant to AstraZeneca, Novartis, Travere Therapeutics, and Mantra Bio; and received grant support from Sana. JB reports research grants from Argenx, Calliditas Therapeutics, Chinook Therapeutics, Galapagos NV, GlaxoSmithKline, Novartis, and Travere Therapeutics; and is medical/scientific advisor to Alnylam Pharmaceuticals, Argenx, Astellas Pharma, BioCryst Pharmaceuticals, Calliditas Therapeutics, Chinook Therapeutics, Dimerix, Galapagos NV, Novartis, Omeros, Travere Therapeutics, Inc., UCB, Vera Therapeutics, and Visterra. HJLH has served as consultant to AbbVie, AstraZeneca, Bayer, Boehringer Ingelheim, Chinook, CSL Pharma, Dimerix, Gilead, Janssen, Merck, Mitsubishi Tanabe, Mundi Pharma, Novo Nordisk, and Travere Therapeutics, Inc. ILN reports receiving honorarium for scientific presentation from AstraZeneca, Bayer, Roche, and Novartis. VP reports receiving grants from Pfizer, which provided study drug and initial seed funding, during the conduct of the study, and grants from AbbVie for a clinical trial steering committee; personal fees from Amgen for serving on an advisory board; serving on a clinical trial steering committee for Astellas; receiving personal fees from AstraZeneca Boehringer Ingelheim, Janssen, Novo Nordisk, and Novartis for serving on a steering committee, advisory committee, and scientific presentations; serving on a trial steering committee and advisory committee for Bayer; receiving personal fees from Chinook Therapeutics for serving on an advisory committee; serving on a data and safety monitoring committee for Dimerix; serving on the board of directors for George Clinical; serving on a steering committee and advisory committee for Gilead, GlaxoSmithKline, and Travere Therapeutics, Inc.; serving on an advisory committee for Medimmune; receiving personal fees from Mitsubishi Tanabe for scientific presentations; receiving personal fees from Mundipharma for advisory committee and scientific presentations; and serving on an advisory committee for Vifor Pharma. BR has served as consultant to Calliditas, Omeros, Novartis, Travere Therapeutics, Inc., and Vera. HTri reports honorarium for scientific work from Alexion, AstraZeneca, Bayer, BioCryst, Calliditas, Chinook, Dimerix, GlaxoSmithKline, Novartis, Omeros, Travere Therapeutics, Inc., Verra Therapeutics, and Visterra Otsuka. MGW reports honorarium for scientific presentation from Amgen, AstraZeneca, Baxter, CSL Behring, Dimerix, Eledon, Otuska, Kira Pharma, Alpine, George Clinical, Horizon, and Travere Therapeutics, Inc. AM has served as consultant to Travere Therapeutics, Inc. through contract with JAMCO Pharma Consulting AB. JI, WR, EM, PWB, SR, SB, and RK are employees and stockholders of Travere Therapeutics, Inc.

## Supplementary Material

Supplementary File (PDF)

**Table S1.** Genes included in the nephrotic syndrome/FSGS panel.

**Table S2.** Demographics, medical history, and baseline clinical characteristics of patients enrolled in DUPLEX by race.

**Table S3.** Pretreatment and baseline medications for patients enrolled in DUPLEX by race.

**Table S4.** Demographics, medical history, and baseline clinical characteristics of patients enrolled in DUPLEX by geographic region.

**Table S5.** Pretreatment and baseline medications for patients enrolled in DUPLEX by geographic regions.

**CONSORT Checklist.**
