# Supplementary material for: Focal Segmental Glomerulosclerosis Patient Baseline Characteristics in the Sparsentan Phase 3 DUPLEX Study
Source: Kidney Int Rep. 2024 Jan 28;9(4):1020–30. doi: 10.1016/j.ekir.2024.01.032 (PMC11101813; doi:10.1016/j.ekir.2024.01.032)
Supplement: Supplementary File (PDF) [file mmc1.pdf]

Supplemental Materials

Supplementary Table S1. Genes included in the nephrotic syndrome/FSGS panel

Official gene symbol	OMIM ID
ACTN4	604638
ANKFY1	607927
ANLN	616027
APOL1	603743
ARHGAP24	610586
ARHGDIA	601925
AVIL	613397
CD2AP	604241
CDK20	610076
COL4A3	120070
COL4A4	120131
COL4A5	303630
COL4A6	303631
COQ2	609825
COQ6	614647
COQ8B	615567
CRB2	609720
CUBN	602997
DAAM2	606627
DGKE	601440
DLC1	604258
EMP2	602334
FAT1	600976
GAPVD1	611714
GON7	617436
INF2	610982

ITGA3	605025
ITGB4	147557
ITSN1	602442
ITSN2	604464
KANK1	607704
KANK2	614610
KANK4	614612
KAT2B	602303
KIRREL1	607428
LAGE3	300060
LAMA5	601033
LAMB2	150325
LMX1B	602575
MAFB	608968
MAGI2	606382
MYH9	160775
MYO1E	601479
NEU1	608272
NFKB2	164012
NPHS1	602716
NPHS2	604766
NUP107	607617
NUP133	607613
NUP160	607614
NUP205	614352
NUP93	614351
OSGEP	610107
PAX2	167409
PDSS2	610564

PLCE1	608414
PODXL	602632
PTPRO	600579
SCARB2	602257
SGPL1	603729
SMARCAL1	606622
TBC1D8B	301027
TNS2	607717
TP53RK	608679
TPRKB	608680
TRIM8	606125
TRPC6	603652
TTC21B	612014
WDR4	605924
WDR73	616144
WT1	607102
XPO5	607845
YRDC	612276

Table shows genes included in the 72-gene nephrotic syndrome/FSGS test panel, with the addition of *PODXL*.

OMIM, Online Mendelian Inheritance in Man.

Supplementary Table S2. Demographics, medical history, and baseline clinical characteristics of patients enrolled in DUPLEX by race

**Characteristic**	**White (*n*=271)**	**Asian (*n*=49)**	**Black or African American (*n*=25)**	**Other (*n*=26)**
Age at informed consent, years	43.0 (28.0-56.0)	45.0 (31.0-56.0)	35.0 (27.0-40.0)	40.5 (20.0-49.0)
Pediatrics (<18 years)	25 (9.2)	1 (2.0)	4 (16.0)	5 (19.2)
Sex				
Male	145 (53.5)	33 (67.3)	10 (40.0)	12 (46.2)
Female	126 (46.5)	16 (32.7)	15 (60.0)	14 (53.8)
Ethnicity				
Not Hispanic or Latino	203 (74.9)	49 (100.0)	19 (76.0)	10 (38.5)
Hispanic or Latino	61 (22.5)	0 (0.0)	5 (20.0)	13 (50.0)
Unknown/not reported	7 (2.6)	0 (0.0)	1 (4.0)	3 (11.5)
Age at FSGS diagnosis, years[b]	37.0 (24.0-52.0)	40.0 (25.0-52.0)	30.0 (19.0-39.0)	32.5 (20.0-45.0)
Time from FSGS diagnosis to informed consent, years[c]	3.0 (1.0-6.0)	2.0 (1.0-6.0)	1.0 (1.0-3.0)	2.0 (0.0-5.0)
History of diabetes and impaired fasting glucose	32 (11.8)	16 (32.7)	5 (20.0)	5 (19.2)
History of hypertension	172 (63.5)	34 (69.4)	18 (72.0)	16 (61.5)
Documented history of nephrotic syndrome[d]	84 (31.0)	12 (24.5)	9 (36.0)	7 (26.9)
Blood pressure, mm Hg, mean ± SD				

Systolic	131.8 ± 14.9	132.0 ± 12.2	132.0 ± 17.3	131.9 ± 17.9
Diastolic	83.4 ± 10.7	86.4 ± 10.8	85.2 ± 8.7	81.5 ± 9.9
BMI, kg/m^2, mean ± SD	27.5 ± 5.9	27.0 ± 4.8	29.9 ± 7.1	28.3 ± 6.1
Edema present	109 (40.2)	18 (36.7)	9 (36.0)	5 (19.2)
Edema grade				
0	162 (59.8)	31 (63.3)	16 (64.0)	21 (80.8)
1	69 (25.5)	14 (28.6)	6 (24.0)	3 (11.5)
2	30 (11.1)	1 (2.0)	2 (8.0)	1 (3.8)
3	10 (3.7)	3 (6.1)	1 (4.0)	1 (3.8)
Hemoglobin, g/l, mean ± SD	135.4 ± 18.2	135.4 ± 18.7	128.0 ± 14.6	128.7 ± 17.8
Plasma lipid profile, mmol/l, mean ± SD				
Total cholesterol	6.3 ± 2.6	5.6 ± 1.8	6.7 ± 2.1	5.5 ±1.4
HDL-C	1.5 ± 0.5	1.4 ± 0.5	1.8 ± 0.7	1.3 ± 0.5
LDL-C	3.7 ± 2.1	3.2 ± 1.5	4.1 ± 1.9	3.2 ± 1.1
Triglycerides	2.4 ± 1.7	2.2 ± 1.1	1.8 ± 0.9	2.3 ± 1.5
Serum potassium, mmol/l, mean ± SD	4.3 ± 0.4	4.3 ± 0.5	4.3 ± 0.5	4.3 ± 0.5
Serum creatinine, µmol/l, mean ± SD	125.1 ± 50.4	126.4 ± 45.0	130.2 ± 56.7	110.0 ± 36.6
Serum albumin, g/l				
Mean ± SD	34.5 ± 7.6	37.6 ± 6.3	33.8 ± 7.2	34.9 ± 7.4
Median (IQR)	36.0 (30.0-40.0)	40.0 (35.0-42.0)	36.0 (28.0-39.0)	37.0 (31.0-41.0)
UP/C, g/g				
Mean ± SD	3.9 ± 2.7	3.2 ± 2.0	3.6 ± 2.2	3.5 ± 1.8
Median (IQR)	3.2 (2.3-4.7)	2.5 (1.8-3.8)	3.2 (1.8-4.1)	2.7 (2.1-5.0)

UA/C, g/g				
Mean ± SD	2.8 ± 1.5	2.4 ± 1.4	2.3 ± 1.0	2.6 ± 1.2
Median (IQR)	2.5 (1.8-3.6)	2.0 (1.5-3.1)	2.4 (1.3-3.1)	2.2 (1.6-3.5)
Nephrotic range UP/C[e]	124 (45.8)	15 (30.6)	9 (36.0)	10 (38.5)
>3.5 g/g in adults (age ≥18 years)	100 (36.9)	14 (28.6)	5 (20.0)	5 (19.2)
>2.0 g/g in pediatrics (age <18 years)	24 (8.9)	1 (2.0)	4 (16.0)	5 (19.2)
eGFR,[f] ml/min/1.73 m^2				
Mean ± SD	63.2 ± 30.8	61.9 ± 25.5	68.5 ± 34.7	68.9 ± 29.1
Median (IQR)	55.0 (40.0-79.0)	54.0 (43.0-79.0)	64.0 (42.0-89.0)	60.5 (50.0-87.0)
eGFR				
≥90 ml/min/1.73 m^2	50 (18.5)	9 (18.4)	6 (24.0)	5 (19.2)
≥60 to <90 ml/min/1.73 m^2	73 (26.9)	10 (20.4)	7 (28.0)	8 (30.8)
≥45 to <60 ml/min/1.73 m^2	52 (19.2)	16 (32.7)	2 (8.0)	9 (34.6)
≥30 to <45 ml/min/1.73 m^2	78 (28.8)	11 (22.4)	8 (32.0)	4 (15.4)
≥15 to <30 ml/min/1.73 m^2	18 (6.6)	3 (6.1)	2 (8.0)	0 (0)

Data are given as n (%) or median (IQR) unless otherwise noted. Patients who selected more than one race are included in Other. The "Other" category includes patients who selected American Indian or Alaska Native, Native Hawaiian or Other Pacific Islander, or Other.

BMI, body mass index; CKD-EPI, CKD Epidemiology Collaboration; eGFR, estimated glomerular filtration rate; FSGS, focal segmental glomerulosclerosis; HbA1c, hemoglobin A1c; HDL-C, high-density lipoprotein cholesterol; LDL-C, low-density lipoprotein cholesterol; UP/C, urine protein/creatinine ratio.

[a]Patients may have selected more than one race. "Other" race included American Indian or Alaska Native, Native Hawaiian or Other Pacific Islander, and Other.

[b]Age at FSGS diagnosis is derived based on the year of FSGS diagnosis and year of birth.

[c]Time from FSGS diagnosis is derived based on the year of FSGS diagnosis and year of signed informed consent.

[d]Documented history of nephrotic syndrome was defined as present if in the medical history or if all of the following conditions were met at any of the visits prior to the first dose of randomized: UP/C >3.5 g/g (adults) or UP/C >2 g/g (pediatrics), serum albumin <3.0 g/dL, and abnormal edema from physical examination. [e]Nephrotic range UP/C based on the cutoff of 3.5 g/g in adults (≥18 years) and 2.0 g/g in pediatrics (<18 years). The percentage with nephrotic range UP/C was calculated based on the number of patients within each age category.

[f]eGFR was determined using the CKD-EPI equation for patients ≥16 years of age at screening, and the modified Schwartz formula for patients <16 years of age at screening.

Supplementary Table S3. Pretreatment and baseline medications for patients enrolled in DUPLEX by race

**Characteristic**	**White (*n*=271)**	**Asian (*n*=49)**	**Black or African American (*n*=25)**	**Other (*n*=26)**
Pretreatment RAASi[a]				
Any RAASi	198 (73.1)	37 (75.5)	19 (76.0)	17 (65.4)
ACEi	109 (40.2)	9 (18.4)	7 (28.0)	8 (30.8)
ARB	111 (41.0)	28 (57.1)	12 (48.0)	12 (46.2)
Aldosterone blockers	20 (7.4)	0 (0)	1 (4.0)	1 (3.8)
Baseline medication use[b]				
Non-RAASi antihypertensive medications	164 (60.5)	29 (59.2)	16 (64.0)	10 (38.5)
Diuretics	107 (39.5)	9 (18.4)	12 (48.0)	3 (11.5)
Beta-blockers	65 (24.0)	18 (36.7)	2 (8.0)	5 (19.2)
Calcium channel blockers	81 (29.9)	21 (42.9)	7 (28.0)	5 (19.2)
Alpha-blockers	35 (12.9)	11 (22.4)	1 (4.0)	2 (7.7)
Other	17 (6.3)	2 (4.1)	3 (12.0)	0 (0)
≥2 antihypertensive medications at baseline	93 (34.3)	20 (40.8)	7 (28.0)	4 (15.4)
Number of antihypertensive medications per patient				
Mean ± SD	1.9 ± 1.0	2.1 ± 1.0	1.6 ± 0.7	1.6 ± 0.8
Median (IQR)	2.0 (1-2)	2.0 (1-2)	1.0 (1-2)	1.0 (1-2)

RAASi[c]	9 (3.3)	1 (2.0)	1 (4.0)	1 (3.8)
Lipid-lowering medications	164 (60.5)	30 (61.2)	11 (44.0)	12 (46.2)
Immunosuppressive agents[d]				
Any immunosuppressive therapy	73 (26.9)	12 (24.5)	5 (20.0)	2 (7.7)
Steroids	38 (14.0)	10 (20.4)	4 (16.0)	1 (3.8)
CNIs (cyclosporine, tacrolimus)	44 (16.2)	6 (12.2)	2 (8.0)	2 (7.7)
ACTH	1 (0.4)	1 (2.0)	0 (0)	0 (0)
Other	1 (0.4)	0 (0)	0 (0)	0 (0)

Data are given as n (%) unless otherwise noted. Patients who selected more than one race are included in Other. The "Other" category includes patients who selected American Indian or Alaska Native, Native Hawaiian or Other Pacific Islander, or Other.

ACEi, angiotensin converting enzyme inhibitor; ACTH, adrenocorticotropic hormone; ARB, angiotensin receptor blocker; CNI, calcineurin inhibitor; RAS, renin-angiotensin system; RAASi, renin-angiotensin-aldosterone system inhibitors.

[a]Pretreatment medications were initiated and stopped prior to the initial dose of study medication.

[b]Baseline medications were started prior to randomization (Day 1) and continued after the initial dose of study medication.

[c]12 patients were on RAASi at baseline, of whom 8 were on RAS inhibitors and 4 were on aldosterone antagonists.

[d]Only immunosuppressive agents for renal indications are included. No patients received mycophenolate mofetil plus azathioprine.

Supplementary Table S4. Demographics, medical history, and baseline clinical characteristics of patients enrolled in DUPLEX by geographic region

**Characteristic**	**North America (*n*=144)**	**Europe (*n*=134)**	**Asia Pacific (*n*=46)**	**South America (*n*=47)**
Age at informed consent, years	33.0 (18.0-55.0)	44.0 (35.0-56.0)	47.0 (36.0-57.0)	40.0 (27.0-52.0)
Adults (≥18 years)	110 (76.4)	133 (99.3)	46 (100.0)	47 (100.0)
Pediatrics (<18 years)	34 (23.6)	1 (0.7)	0 (0.0)	0 (0.0)
Sex				
Male	77 (53.5)	71 (53.0)	31 (67.4)	21 (44.7)
Female	67 (46.5)	63 (47.0)	15 (32.6)	26 (55.3)
Race[a]				
White	100 (69.4)	122 (91.0)	9 (19.6)	40 (85.1)
Black or African American	16 (11.1)	4 (3.0)	0 (0.0)	5 (10.6)
Asian	11 (7.6)	2 (1.5)	36 (78.3)	0 (0.0)
Other	17 (11.8)	6 (4.5)	1 (2.2)	2 (4.3)
Ethnicity				
Not Hispanic or Latino	108 (75.0)	122 (91.0)	44 (95.7)	7 (14.9)
Hispanic or Latino	34 (23.6)	8 (6.0)	1 (2.2)	36 (76.6)
Not reported	1 (0.7)	2 (1.5)	1 (2.2)	3 (6.4)
Unknown	1 (0.7)	2 (1.5)	0 (0.0)	1 (2.1)
Age at FSGS diagnosis, years[b]	31.0 (16.0-51.0)	39.5 (27.0-52.0)	41.5 (33.0-56.0)	37.0 (22.0-48.0)

Time from FSGS diagnosis to informed consent, years[c]	2.0 (1.0-4.0)	3.0 (1.0-8.0)	3.0 (1.0-7.0)	3.0 (1.0-7.0)
History of diabetes and impaired fasting glucose	26 (18.1)	13 (9.7)	14 (30.4)	5 (10.6)
History of hypertension	98 (68.1)	85 (63.4)	32 (69.6)	25 (53.2)
Documented history of nephrotic syndrome[d]	54 (37.5)	32 (23.9)	10 (21.7)	16 (34.0)
Blood pressure, mm Hg, mean ± SD				
Systolic	129.9 ± 16.1	134.1 ± 14.5	133.6 ± 13.1	129.8 ± 13.4
Diastolic	81.8 ± 11.5	84.9 ± 9.6	86.5 ± 10.8	84.5 ± 9.0
BMI, kg/m^2, mean ± SD	28.4 ± 6.9	27.1 ± 5.3	26.7 ± 4.5	27.8 ± 5.1
Edema present	52 (36.1)	51 (38.1)	17 (37.0)	21 (44.7)
Edema grade				
0	92 (63.9)	83 (61.9)	29 (63.0)	26 (55.3)
1	36 (25.0)	30 (22.4)	11 (23.9)	15 (31.9)
2	11 (7.6)	18 (13.4)	2 (4.3)	3 (6.4)
3	5 (3.5)	3 (2.2)	4 (8.7)	3 (6.4)
4	0 (0.0)	0 (0.0)	0 (0.0)	0 (0.0)
Hemoglobin, g/l, mean ± SD	130.3 ± 17.7	137.4 ± 17.1	135.4 ± 20.4	137.3 ± 17.9
Plasma lipid profile, mmol/l, mean ± SD				
Total cholesterol	6.1 ± 2.2	6.3 ± 2.6	5.5 ± 2.6	6.9 ± 2.2
HDL cholesterol	1.4 ± 0.5	1.6 ± 0.5	1.4 ± 0.4	1.5 ± 0.6
LDL cholesterol	3.6 ± 1.7	3.6 ± 2.1	3.1 ± 2.2	4.1 ± 1.8
Triglycerides	2.4 ± 1.8	2.3 ± 1.5	2.1 ± 1.3	2.6 ± 1.5

Serum potassium, mmol/l, mean ± SD	4.3 ± 0.5	4.3 ± 0.4	4.3 ± 0.5	4.3 ± 0.4
Serum creatinine, μmol/l, mean ± SD	121.9 ± 50.9	125.7 ± 47.7	127.0 ± 42.1	126.9 ± 56.3
Serum albumin, g/l				
Mean ± SD	33.9 ± 7.8	34.9 ± 7.0	38.1 ± 5.6	34.7 ± 8.5
Median (IQR)	35.5 (29.0-40.0)	36.0 (31.0-39.0)	40.0 (36.0-42.0)	36.0 (27.0-41.0)
UP/C, g/g				
Mean ± SD	4.2 ± 3.1	3.7 ± 2.1	2.9 ± 1.9	3.2 ± 1.9
Median (IQR)	3.3 (2.4-5.1)	3.2 (2.3-4.8)	2.5 (1.8-3.5)	2.9 (1.9-4.0)
Nephrotic range UP/C[e]	76 (52.8)	54 (40.3)	12 (26.1)	16 (34.0)
Nephrotic range UP/C >3.5 g/g in adults (age ≥18 years)	43 (29.9)	53 (39.6)	12 (26.1)	16 (34.0)
Nephrotic range UP/C >2.0 g/g in pediatrics (age <18 years)	33 (22.9)	1 (0.7)	0 (0)	0 (0)
eGFR,[f] ml/min/1.73 m^2				
Mean ± SD	67.3 ± 35.6	61.4 ± 27.6	59.6 ± 22.7	63.9 ± 25.3
Median (IQR)	57.0 (40.5-84.5)	54.5 (41.0-79.0)	53.5 (43.0-74.0)	70.0 (42.0-84.0)
eGFR				
≥90 ml/min/1.73 m^2	33 (22.9)	21 (15.7)	7 (15.2)	9 (19.1)
≥60 to <90 ml/min/1.73 m^2	35 (24.3)	37 (27.6)	9 (19.6)	17 (36.2)
≥45 to <60 ml/min/1.73 m^2	27 (18.8)	29 (21.6)	17 (37.0)	6 (12.8)
≥30 to <45 ml/min/1.73 m^2	42 (29.2)	37 (27.6)	10 (21.7)	12 (25.5)

≥15 to <30 ml/min/1.73 m^2	7 (4.9)	10 (7.5)	3 (6.5)	3 (6.4)

Data are given as n (%) or median (IQR) unless otherwise noted.

FSGS, focal segmental glomerulosclerosis; HbA1c, hemoglobin A1c; SD, standard deviation; UP/C, urine protein/creatinine ratio.

[a]Patients may have selected more than one race. "Other" race included American Indian or Alaska Native, Native Hawaiian or Other Pacific Islander, and Other.

[b]Age at FSGS diagnosis is derived based on the year of FSGS diagnosis and year of birth.

[c]Time from FSGS diagnosis is derived based on the year of FSGS diagnosis and year of signed informed consent.

[d]Documented history of nephrotic syndrome was defined as present if in the medical history or if all of the following conditions were met at any of the visits prior to the first dose of randomized: UP/C >3.5 g/g (adults) or UP/C >2 g/g (pediatrics), serum albumin <3.0 g/dL, and abnormal edema from physical examination. [e]Nephrotic range UP/C based on the cutoff of 3.5 g/g in adults (≥18 years) and 2.0 g/g in pediatrics (<18 years). The percentage with nephrotic range UP/C within each age category was calculated using the overall number of adult or pediatric patients (as appropriate) as denominator.

[f]eGFR was determined using the CKD-EPI equation for patients ≥16 years of age at screening, and the modified Schwartz formula for patients <16 years of age at screening.

Supplementary Table S5. Pretreatment and baseline medications for patients enrolled in DUPLEX by geographic regions

**Characteristic**	**North America (*n*=144)**	**Europe (*n*=134)**	**Asia Pacific (*n*=46)**	**South America (*n*=47)**
Pretreatment RAASi[a]				
Any RAASi	111 (77.1)	90 (67.2)	36 (78.3)	34 (72.3)
ACEi	52 (36.1)	55 (41.0)	10 (21.7)	16 (34.0)
ARB	64 (44.4)	52 (38.8)	26 (56.5)	21 (44.7)
Aldosterone blockers	6 (4.2)	12 (9.0)	0 (0.0)	4 (8.5)
Baseline medication use[b]				
Non-RAASi antihypertensive medications	79 (54.86)	84 (62.69)	28 (60.87)	28 (59.57)
Diuretics	47 (32.6)	56 (41.8)	8 (17.4)	20 (42.6)
Beta-blockers	35 (24.3)	27 (20.1)	17 (37.0)	11 (23.40)
Calcium channel blockers	44 (30.6)	41 (30.6)	17 (37.0)	12 (25.5)
Alpha-blockers	17 (11.8)	18 (13.4)	9 (19.6)	5 (10.6)
Other	5 (3.5)	12 (9.0)	3 (6.5)	2 (4.3)
≥2 antihypertensive medications at baseline (excluding RAASi medications)	49 (34.0)	44 (32.8)	17 (37.0)	14 (29.8)
Number of antihypertensive medications per patient (including RAASi medications)				
Mean ± SD	2.0 ± 1.0	1.9 ± 1.1	1.9 ± 0.9	1.8 ± 0.9
Median (IQR)	2.0 (1-2)	2.0 (1-2)	2.0 (1-2)	1.5 (1-3)

RAASi[c]	3 (2.1)	7 (5.2)	1 (2.2)	1 (2.1)
Lipid-lowering medications	74 (51.4)	86 (64.2)	30 (65.2)	27 (57.5)
Immunosuppressive agents[d]				
Any immunosuppressive therapy	41 (28.5)	30 (22.4)	11 (23.9)	10 (21.3)
Steroids	18 (12.5)	17 (12.7)	11 (23.9)	7 (14.9)
CNIs (cyclosporine, tacrolimus)	27 (18.8)	16 (11.9)	5 (10.9)	6 (12.8)
ACTH	2 (1.4)	0 (0.0)	0 (0.0)	0 (0.0)
Other	1 (0.7)	0 (0.0)	0 (0.0)	0 (0.0)

Data are given as n (%) unless otherwise noted.

ACEi, angiotensin converting enzyme inhibitor; ACTH, adrenocorticotropic hormone; ARB, angiotensin receptor blocker; CNI, calcineurin inhibitor; RAS, renin-angiotensin system; RAASi, renin-angiotensin-aldosterone system inhibitors.

[a]Pretreatment medications were initiated and stopped prior to the initial dose of study medication.

[b]Baseline medications were started prior to randomization (Day 1) and continued after the initial dose of study medication.

[c]12 patients were on RAASi at baseline, of whom 8 were on RAS inhibitors and 4 were on aldosterone antagonists.

[d]Only immunosuppressive agents for renal indications are included. No patients received mycophenolate mofetil plus azathioprine.

CONSORT 2010 checklist of information to include when reporting a randomised trial*

Section/Topic	Item No	Checklist item	Reported on page No
**Title and abstract**			
	1a	Identification as a randomised trial in the title	Not in title as this is an aggregated baseline characteristics paper
	1b	Structured summary of trial design, methods, results, and conclusions (for specific guidance see CONSORT for abstracts)	3
**Introduction**			
Background and objectives	2a	Scientific background and explanation of rationale	4-5
	2b	Specific objectives or hypotheses	5
**Methods**			
Trial design	3a	Description of trial design (such as parallel, factorial) including allocation ratio	6 (allocation ration not included due to baseline aggregated focus)
	3b	Important changes to methods after trial commencement (such as eligibility criteria), with reasons	NA
Participants	4a	Eligibility criteria for participants	6
	4b	Settings and locations where the data were collected	6
Interventions	5	The interventions for each group with sufficient details to allow replication, including how and when they were actually administered	NA (baseline focus)
Outcomes	6a	Completely defined pre-specified primary and secondary outcome measures, including how and when they were assessed	7
	6b	Any changes to trial outcomes after the trial commenced, with reasons	NA (baseline focus)
Sample size	7a	How sample size was determined	NA (baseline focus; study design paper is cited)
	7b	When applicable, explanation of any interim analyses and stopping guidelines	NA (baseline focus; study design paper is cited)
Randomisation:			
	8a	Method used to generate the random allocation sequence	NA (baseline focus)

Sequence generation	8b	Type of randomisation; details of any restriction (such as blocking and block size)	NA (baseline focus)
Allocation concealment mechanism	9	Mechanism used to implement the random allocation sequence (such as sequentially numbered containers), describing any steps taken to conceal the sequence until interventions were assigned	NA (baseline focus)
Implementation	10	Who generated the random allocation sequence, who enrolled participants, and who assigned participants to interventions	NA (baseline focus)
Blinding	11a	If done, who was blinded after assignment to interventions (for example, participants, care providers, those assessing outcomes) and how	6
	11b	If relevant, description of the similarity of interventions	6
Statistical methods	12a	Statistical methods used to compare groups for primary and secondary outcomes	NA (baseline focus)
	12b	Methods for additional analyses, such as subgroup analyses and adjusted analyses	NA (baseline focus)
**Results**			
Participant flow (a diagram is strongly recommended)	13a	For each group, the numbers of participants who were randomly assigned, received intended treatment, and were analysed for the primary outcome	NA (baseline focus; baseline characteristics are aggregated)
	13b	For each group, losses and exclusions after randomisation, together with reasons	NA (baseline focus; baseline characteristics are aggregated)
Recruitment	14a	Dates defining the periods of recruitment and follow-up	NA (trial in progress)
	14b	Why the trial ended or was stopped	NA (trial in progress)
Baseline data	15	A table showing baseline demographic and clinical characteristics for each group	26-30 (aggregated data)
Numbers analysed	16	For each group, number of participants (denominator) included in each analysis and whether the analysis was by original assigned groups	NA (baseline focus)
Outcomes and estimation	17a	For each primary and secondary outcome, results for each group, and the estimated effect size and its precision (such as 95% confidence interval)	NA (baseline focus)
	17b	For binary outcomes, presentation of both absolute and relative effect sizes is recommended	NA (baseline focus)
Ancillary analyses	18	Results of any other analyses performed, including subgroup analyses and adjusted analyses, distinguishing pre-specified from exploratory	11-12 and Supplemental tables S2-S5
Harms	19	All important harms or unintended effects in each group (for specific guidance see CONSORT for harms)	NA (baseline focus)
**Discussion**			
Limitations	20	Trial limitations, addressing sources of potential bias, imprecision, and, if relevant, multiplicity of analyses	17 (baseline focus)

Generalisability	21	Generalisability (external validity, applicability) of the trial findings	12-14; 17-18 (baseline focus)
Interpretation	22	Interpretation consistent with results, balancing benefits and harms, and considering other relevant evidence	12-16
**Other information**			
Registration	23	Registration number and name of trial registry	6
Protocol	24	Where the full trial protocol can be accessed, if available	NA (trial ongoing)
Funding	25	Sources of funding and other support (such as supply of drugs), role of funders	19

*We strongly recommend reading this statement in conjunction with the CONSORT 2010 Explanation and Elaboration for important clarifications on all the items. If relevant, we also recommend reading CONSORT extensions for cluster randomised trials, non-inferiority and equivalence trials, non-pharmacological treatments, herbal interventions, and pragmatic trials. Additional extensions are forthcoming: for those and for up to date references relevant to this checklist, see www.consort-statement.org.
